# Regulation of Splicing Factors by Alternative Splicing and NMD Is Conserved between Kingdoms Yet Evolutionarily Flexible

**DOI:** 10.1093/molbev/msv002

**Published:** 2015-03-03

**Authors:** Liana F. Lareau, Steven E. Brenner

**Affiliations:** ^1^Departments of Molecular and Cell Biology and Plant and Microbial Biology, University of California, Berkeley; ^2^Department of Biochemistry, Stanford University School of Medicine

**Keywords:** alternative splicing, nonsense mediated decay, ultraconserved elements

## Abstract

Ultraconserved elements, unusually long regions of perfect sequence identity, are found in genes encoding numerous RNA-binding proteins including arginine-serine rich (SR) splicing factors. Expression of these genes is regulated via alternative splicing of the ultraconserved regions to yield mRNAs that are degraded by nonsense-mediated mRNA decay (NMD), a process termed unproductive splicing (Lareau et al. 2007; Ni et al. 2007). As all human SR genes are affected by alternative splicing and NMD, one might expect this regulation to have originated in an early SR gene and persisted as duplications expanded the SR family. But in fact, unproductive splicing of most human SR genes arose independently (Lareau et al. 2007). This paradox led us to investigate the origin and proliferation of unproductive splicing in SR genes. We demonstrate that unproductive splicing of the splicing factor SRSF5 (SRp40) is conserved among all animals and even observed in fungi; this is a rare example of alternative splicing conserved between kingdoms, yet its effect is to trigger mRNA degradation. As the gene duplicated, the ancient unproductive splicing was lost in paralogs, and distinct unproductive splicing evolved rapidly and repeatedly to take its place. SR genes have consistently employed unproductive splicing, and while it is exceptionally conserved in some of these genes, turnover in specific events among paralogs shows flexible means to the same regulatory end.

## Introduction

Evolution has provided diverse and sometimes startling ways to regulate gene expression, taking advantage of essentially every step from the birth to the death of an mRNA (Moore 2005). Regulation can impose strong constraints on sequence evolution; the most pronounced sequence constraints have resulted in ultraconserved DNA elements, long regions of at least 200 nt of perfect identity between human and rodent genomes (Bejerano et al. 2004). This level of sequence conservation is rarely observed; there are only several hundred such elements in the human genome, many functioning as transcription enhancers. However, ultraconserved elements are also found within genes encoding numerous RNA-binding proteins (Bejerano et al. 2004, 2006) and they have been shown to play a role in posttranscriptional regulation of these genes (Lareau et al. 2007; Ni et al. 2007).

Among the RNA-binding proteins with ultraconserved elements are arginine-serine rich (SR) proteins, a key family of splicing factors that are involved in alternative and constitutive splicing, mRNA export, and translation (Shepard and Hertel 2009). Surprisingly, the ultraconserved sequence does not encode protein; rather, it is involved in posttranscriptional regulation. All 11 human SR genes are alternatively spliced to produce mRNAs with early stop codons, which are degraded by nonsense-mediated mRNA decay (NMD) rather than producing protein (Lareau et al. 2007; Ni et al. 2007). This process, known as unproductive splicing, downregulates expression by shunting a fraction of a gene’s pre-mRNA into a decay pathway. Unproductive splicing of some SR genes involves an alternative exon with an early in-frame stop codon, termed a poison cassette exon. Ultraconserved elements overlap poison cassette exons in four SR genes, and the poison cassette exons of other SR genes also have exceptional sequence identity between human and mouse.

SR proteins are abundant—some are within the top 2% most translated genes (Ingolia et al. 2011)—and their expression is tightly regulated. In some cases, their unproductive splicing is used as a means of negative autoregulation, perhaps to maintain homeostasis of protein levels. Some SR proteins can bind their own pre-mRNAs and promote unproductive splicing events (Jumaa and Nielsen 1997; Lejeune et al. 2001; Sureau et al. 2001); SRSF4, a gene we investigate in this study, was shown to bind its own pre-mRNA and autoregulate its protein levels (Änkö et al. 2010, 2012). SRSF1 (ASF/SF2) is autoregulated at multiple steps in its expression including splicing and translation (Sun et al. 2010). It is a proto-oncogene, and its unproductive splicing is implicated in the decision to enter the epithelial-to-mesenchymal transition (Karni et al. 2007; Valacca et al. 2010). Unproductive splicing of some SR genes is also cross-regulated by other SR proteins; SRSF3 (SRp20) regulates its own unproductive splicing as well as the unproductive splicing of *SRSF5* and other splicing factor genes (Änkö et al. 2012).

Unproductive splicing affects dozens of unrelated RNA-processing genes including splicing factors, hnRNPs, and core spliceosome components in mammals, *Caenorhabditis elegans*, and plants (Wollerton et al. 2004; Kalyna et al. 2006, 2012; Boutz et al. 2007; Lareau et al. 2007; Ni et al. 2007; Spellman et al. 2007; Barberan-Soler and Zahler 2008; Saltzman et al. 2008, 2011; Barberan-Soler et al. 2009; Palusa and Reddy 2010; Verbeeren et al. 2010); reviewed in McGlincy and Smith (2008). These functionally related but nonhomologous genes must have evolved this regulation independently, suggesting that RNA-binding proteins may readily acquire this form of posttranscriptional regulation. Independent origins of unproductive splicing have also been found in plant splicing factor genes; some instances are conserved throughout the plant kingdom and are associated with highly conserved sequence motifs (Kalyna et al. 2006; Palusa and Reddy 2010; Verbeeren et al. 2010; Rauch et al. 2014). The SR proteins comprise a family of highly similar proteins that expanded through a series of duplications (Busch and Hertel 2011; Richardson et al. 2011). Surprisingly, unproductive splicing has arisen independently even among closely related SR genes; poison cassette exons are found in nonhomologous positions in different SR genes (Lareau et al. 2007).

Ultraconservation reflects recent high selective pressure among mammalian genomes, but it need not imply deep conservation across phyla, and in fact most ultraconserved elements originated within the vertebrates (Stephen et al. 2008). The presence of unproductive splicing in so many functionally related proteins suggests that this regulation is highly advantageous or very easily evolved. But facile evolution and extremely high sequence constraints seem at odds with each other. To resolve this seeming paradox, we must trace the SR proteins’ evolutionary history and determine when each SR gene diverged, whether ancestral SR genes were regulated by unproductive splicing, and how the regulatory system has changed across evolution.

## Results

**Fig. 1.**
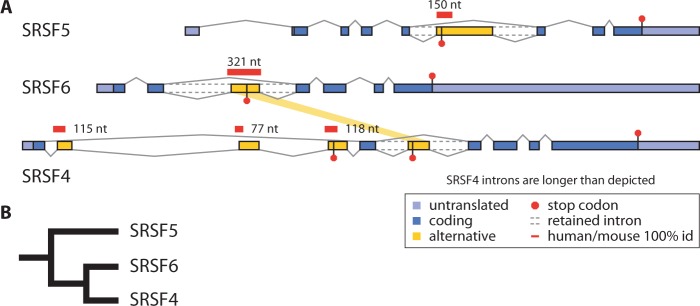
(*A*) Unproductive splicing of the closely related human SR genes *SRSF4*, *SRSF5*, and *SRSF6*. Alternative exons with in-frame stop codons target mRNAs for NMD. Alternative exons in intron 2 of *SRSF6* and *SRSF4* are homologous, shown by yellow band. Regions of 100% sequence identity between human and mouse are indicated. (*B*) Phylogenetic tree of *SRSF4*, *SRSF5*, and *SRSF6*. A single origin of unproductive splicing at the base of this tree would result in homologous events in each gene, but the events observed in SR genes are not homologous (Lareau et al. 2007).

We have conducted a case study of the evolution of unproductive splicing in three related SR genes: *SRSF4* (SRp75), *SRSF5* (SRp40), and *SRSF6* (SRp55). Cassette exons in all three human genes have been shown to trigger NMD when included (fig. 1); when NMD was inhibited in HeLa cells, the exon-included mRNAs were dramatically stabilized, representing between 40% and 70% of the spliced mRNA from each gene (Lareau et al. 2007). We show here that these three genes provide a particularly clear example of the repeated emergence of unproductive splicing. They also include a case of shared unproductive splicing: The closely related human genes *SRSF4* and *SRSF6* have a homologous poison cassette exon within the second intron that was presumably found in the common ancestor gene before duplication (Lareau et al. 2007).

**Fig. 2.**
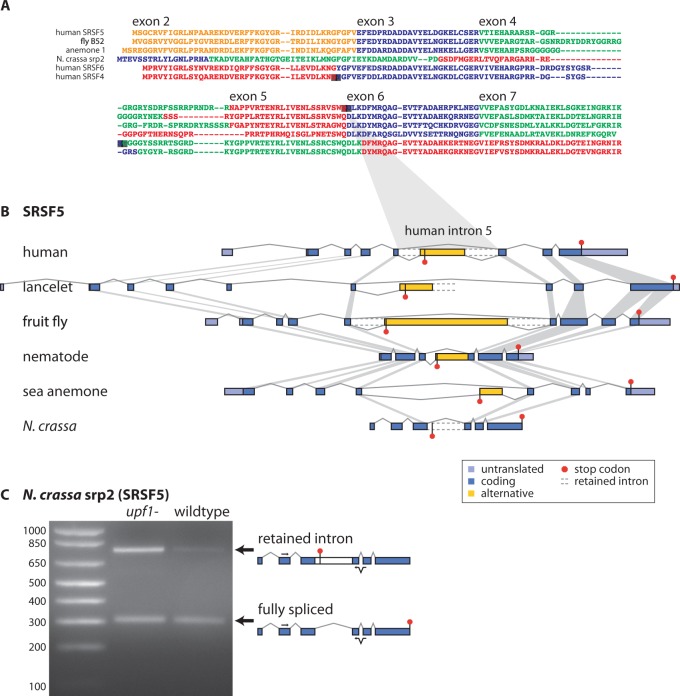
(*A*) Alignment of SRSF4, SRSF5, and SRSF6 protein sequences indicating exon positions. Colors denote exons (labeled relative to SRSF5). Positions of human cassette exon inclusion are indicated with gray boxes at splice junctions. Sequences were trimmed after the second RNA recognition motif (RRM) domain before alignment. See alignment of all genes in supplementary figure S1*B*, Supplementary Material online. (*B*) The exon/intron structure of *SRSF5* is conserved in animals and some intron positions are conserved between animals and fungi. Gray bars show corresponding exons with conserved boundaries. An alternative exon in *SRSF5* is conserved in animals. Intron retention is shared between animals and fungi. *SRSF5* genes in vertebrates including mouse, rat, chicken, frog, and zebrafish were equivalent to human *SRSF5* (supplementary figs. S1 and S2 and table S1, Supplementary Material online). (*C*) mRNAs of *Neurospora crassa srp2* with retained intron 3 (the equivalent of human *SRSF5* intron 5) are stabilized in an NMD-deficient strain.

**Fig. 3.**
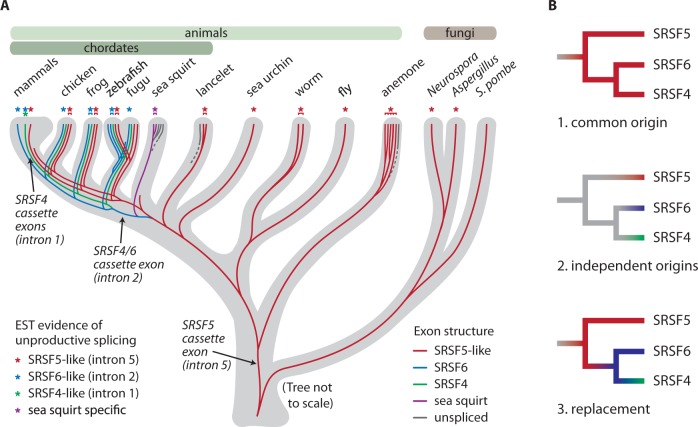
(*A*) Evolution of the *SRSF4/SRSF5/SRSF6* family and origins of its unproductive splicing. A single *SRSF5*-like gene in the ancestor of animals and fungi duplicated in chordates and again in vertebrates. A phylogenetic tree of orthologs was constructed using maximum likelihood and refined by comparing exon boundaries of each gene (supplementary figs. S1*A* and S1*B*, Supplementary Material online). Gray tree shows species relationships and lines show gene relationships (not to scale). Dashed lines indicate uncertain relationships. Asterisks depict genes with EST evidence of unproductive splicing. (*B*) Our data rule out two simple evolutionary models: 1) a single origin of unproductive splicing in the common ancestor of the three genes (shown in red), or 2) independent evolution of unproductive splicing in each gene (shown in red, blue, and green). Instead, the data support a third model: Unproductive splicing was present in the common ancestor, and as the genes duplicated and diverged, unproductive splicing events were replaced with functionally similar unproductive splicing.

To examine the history of alternative splicing in *SRSF4*, *SRSF5*, and *SRSF6*, we first identified their orthologs within the genomes of animals and fungi using detailed phylogenetic analyses (fig. 2; supplementary tables S1 and S2, Supplementary Material online; Barbosa-Morais et al. 2006). A sequence-based phylogenetic tree separated members of the SRSF4/5/6 subfamily from other SR proteins, but because of their high sequence similarity, the tree could not accurately separate orthologs of each protein nor reveal deep ancestry (supplementary fig. S1*A*, Supplementary Material online). Additional information about their relationships was found in the exon/intron structure of each gene (fig. 2*A*; supplementary fig. S1*B*, Supplementary Material online). Introns were added and lost during evolution, and the presence of an intron at a given position can be treated as a phylogenetic character (Henricson et al. 2010). Based on this additional information, we determined which genes were orthologs of *SRSF4*, *SRSF5*, and *SRSF6* in each species, and where the duplications occurred that expanded this subfamily from a single ancestral gene (fig. 3*A*).

Because the exon/intron structures of SR genes have changed over time, the intron positions identify where unproductive splicing could have originated in each gene, via a new poison cassette exon within a particular intron. We inferred that the single member of the group in ancestral animals had an exon/intron structure essentially identical to human SRSF5. Copies of this gene with remarkably conserved exon structure were present in essentially all animals from sea anemone to human (fig. 2*B*). Indeed, intron positions revealed that *B52*, the sole gene in this subfamily in *Drosophila melanogaster*, more closely resembled mammalian *SRSF5* (SRp40) despite its annotation as *SRSF6* (SRp55) in previous studies (Ring and Lis 1994). The single member of the subfamily in fungi, *srp2*, also had very similar exon structure. Therefore, the single ancestor of *SRSF4*, *SRSF5*, and *SRSF6* in opisthokonts was similar to *SRSF5*, and existed predominantly in that form until the chordate lineage.

To find the phylogenetic range of unproductive splicing in *SRSF5*, we compared expressed sequence tags (ESTs) to the *SRSF5* orthologs in each species. We found splicing of an alternative exon in homologous positions in the equivalent of human intron 5 in orthologs of *SRSF5* from vertebrates (human, mouse, rat, chicken, frog, and zebrafish), lancelet, sea urchin, fly, worm, and anemone (fig. 2*B*; supplementary table S1, Supplementary Material online). In many species, we also observed transcripts with introns retained adjacent to the alternative exon, as we had previously noted in numerous human SR genes (Lareau et al. 2007). The intron retention could be directly regulated; or, complex regulation of the alternative exon could cause particularly slow splicing of these introns, leading to accumulation of partially processed transcripts.

We examined the transcripts produced from alternative splicing occurring at homologous positions in *SRSF5* orthologs (supplementary table S1 and fig. S2, Supplementary Material online). In all species studied here, the cassette exons or retained introns introduced early stop codons expected to lead to NMD. In frog and zebrafish, we also found single mRNAs with early polyadenylation in the alternative exon, predicted to evade NMD and produce truncated protein; early polyadenylation might compete with splicing of the downstream exons for different regulatory outcomes. In human cells, *SRSF5* alternative splicing was confirmed to elicit NMD (Lareau et al. 2007), and our reanalysis of a screen for NMD targets in *D. melanogaster* revealed that an isoform of *B52* was indeed stabilized upon inhibition of NMD (Hansen et al. 2009). Thus, unproductive splicing of *SRSF5* orthologs, via alternative splicing within the equivalent of human intron five, is essentially universal across the metazoa.

Remarkably, we also found that ESTs from ascomycete and basidiomycete fungi including *Neurospora cras*s*a*, *Aspergillus niger*, and *Postia placenta* showed retention of the same intron that is alternatively spliced or retained in metazoan *SRSF5* (fig. 2*B*). The retained introns contained in-frame stop codons, leading us to test whether the *N. crassa* mRNA was degraded by NMD. The alternative splice form was dramatically stabilized in a strain lacking *nam7*, ortholog of the NMD factor Upf1 (fig. 2*C*). The assay was designed to amplify only processed mRNAs, not unspliced pre-mRNA, by requiring that one polymerase chain reaction (PCR) primer span a splice junction and by amplifying a region including multiple exons. Thus, the *N. crassa* ortholog of *SRSF5* is frequently spliced into a specific mature mRNA, retaining an intron in a position equivalent to human intron five, that is a target of NMD. Srp2*,* the *Schizosaccharomyces pombe* ortholog of SRSF5, can bind a splicing enhancer in its own pre-mRNA, suggesting that autoregulation of this SR protein may occur in fungi as well as metazoa (Webb et al. 2005). Our results show that a specific unproductive splicing event in *SRSF5*—retention of NMD-inducing intron five—is conserved between the animal and fungi kingdoms.

The gene structure and alternative splicing of *SRSF5* were remarkably static in metazoa and fungi, across an evolutionary distance of over 1 billion years, but a more dynamic scene developed among chordate-specific paralogs. We inferred that *SRSF6* resulted from a duplication of *SRSF5* in chordates, and *SRSF4* arose from a subsequent duplication of *SRSF6* in vertebrates. The gene structure changed dramatically after the first duplication; no intron positions are conserved between *SRSF5* and *SRSF4/SRSF6* (fig. 2*A*). In particular, the intron that contains the conserved poison cassette exon in *SRSF5* is not present in *SRSF4/SRSF6* and thus the original unproductive splicing was necessarily lost when the gene structure changed after duplication.

The ancestral unproductive splicing seen in *SRSF5* was lost in *SRSF6*, concomitant with gene structure changes that eliminated the intron that had contained the poison cassette exon. However, we identified ESTs in many vertebrates showing distinct unproductive splicing in a different, vertebrate-specific intron of *SRSF6* (human intron 2), suggesting that it arose within vertebrates. We observed this alternative exon in the second intron of *SRSF6* in vertebrates from fish to mammals, including human, mouse, rat, dog, chicken, frog, and fish (fig. 3*A*; supplementary table S2, Supplementary Material online). Homologous unproductive splicing was seen in the same position in the close paralog, *SRSF4*, in human and frog ESTs (fig. 3*A*; supplementary table S2, Supplementary Material online). These exons in human *SRSF4* and *SRSF6* were confirmed to elicit NMD in human cells (Lareau et al. 2007). We concluded that, in a chordate or early vertebrate, the single precursor of *SRSF6* and *SRSF4* (derived from duplication of *SRSF5*) lost its original *SRSF5*-like unproductive splicing due to gene rearrangements and gained a new alternative exon with the same effect to elicit NMD.

**Fig. 4.**
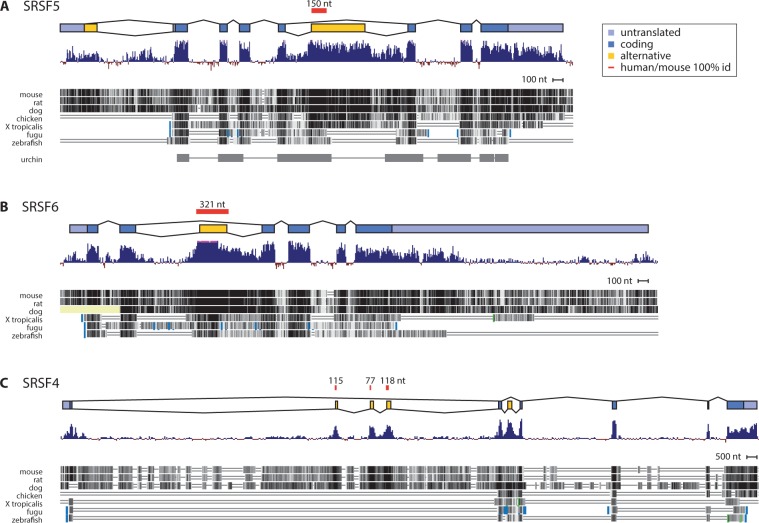
Conservation of SRSF4, SRSF5, and SRSF6 was assessed using data from vertebrate PhyloP conservation scores and whole-genome alignment tracks in the UCSC human genome browser (Pollard et al. 2010; Fujita et al. 2011). Regions of human/mouse 100% identity in alternative exons are marked with red bars. Vertebrate alignments are from MultiZ alignment of 46 vertebrates against human genome version hg19 and sea urchin alignment is from *Strongylocentrotus purpuratus* (September 2006 [Baylor 2.1/strPur2]) net alignment against human genome version hg18. The alternative exons of (*A*) SRSF5 and (*B*) SRSF6 have detectable similarity in all vertebrates and SRSF5 also has detectable homology in sea urchin. (*C*) Intron 1 of SRSF4, including three cassette exons, has no detectable similarity outside of mammals.

The second-intron cassette exon is ultraconserved in *SRSF6* but less conserved in *SRSF4*, suggesting relaxed selective pressure in *SRSF4* (fig. 4), and the exon appears in ESTs from fewer species. After the second duplication and slight divergence of *SRSF4*, the cassette exon may have been lost in SRSF4 in many vertebrate lineages, or it may be spliced at low levels that are less likely to be detected.

Finally, in mammals only, we observed ESTs showing yet another, distinct unproductive splicing event in the first intron of *SRSF4*, suggesting that this additional splice form evolved recently. This splice form, with three coordinate cassette exons in a very long first intron, is seen in human, mouse, and rat ESTs (supplementary table S2, Supplementary Material online). The extended first intron is seen in mammalian *SRSF4* genes but not other vertebrates (fig. 4*C*), and the intron contains many Alu repeats and other mammal-specific repetitive elements. Inhibition of NMD in human cells confirmed that these cassette exons are included at high levels and elicit NMD (Lareau et al. 2007), and the cassette exon sequences are highly conserved among mammals (fig. 4*C*). Although this unproductive splicing appears specific to *SRSF4* and limited to mammals, we cannot rule out the possibility that it originated in the ancestor of *SRSF4* and *SRSF6* and was lost in many lineages.

Our results disagree with two simple evolutionary models: 1) a single origin of unproductive splicing in all three genes, or 2) independent evolution of similar unproductive splicing in each gene (homoplasy). Instead, the data support a third model: Ancestral unproductive splicing was replaced or partially replaced by new unproductive splicing after each duplication (fig. 3*B*).

Most human ultraconserved sequences originated in land vertebrates and were under recent stronger selection in amniotes, but the ultraconserved elements in splicing-related proteins are generally older (Stephen et al. 2008). We have found detectable sequence similarity, based on UCSC whole-genome alignments (Fujita et al. 2011), to the human *SRSF5* alternative exon in all vertebrates and even in the sea urchin, a deuterostome with a pre-Cambrian common ancestor (fig. 4*A*). The alternative exon of *SRSF6* first appeared in vertebrates, but despite its more recent origin, it is ultraconserved in mammals and under relatively uniform selection in all vertebrates (fig. 4*B*). This process is also evident much more recently in *SRSF4*, whose mammalian-specific cassette exons are as conserved among mammals as its coding exons despite much more recent origins (fig. 4*C*). The patterns of sequence conservation indicate that essentially as soon as the function of each alternative exon evolved, its sequence was subject to strong selective pressure.

## Discussion

It has become clear that unproductive splicing regulates a broad and diverse group of RNA-binding proteins in species from plants to humans. Many of these proteins autoregulate their expression by binding their own pre-mRNAs and promoting their processing into unproductive mRNAs. Why is this seemingly wasteful mechanism so pervasive? In cases where autoregulation would be beneficial, perhaps to maintain homeostasis of protein levels, unproductive splicing is an evolutionarily accessible mechanism for autoregulation of proteins that already have RNA-binding capability. Autoregulation requires the means to measure concentration and a mechanism to act on that concentration. RNA is an intrinsic sensor of RNA-binding protein concentration, and the proteins have a native ability to affect processing of their targets. Regulatory unproductive splicing could then evolve by a simple process such as creation of a new poison cassette exon within an existing intron. The exon needs only an in-frame stop codon and binding sites for the core spliceosome and splicing regulators; it would be under no protein-coding sequence constraint. These relatively minimal requirements may explain how unproductive splicing has evolved again and again in unrelated RNA-binding proteins.

In the SR genes examined here, we observed loss and replacement of unproductive splicing events after duplications, rather than recent evolution of analogous regulation in each individual gene (fig. 3*B*). Immediately after gene duplication, negative feedback can maintain protein levels despite higher gene dosage. Initially, the identical copies would be both auto- and cross-regulated, but as one paralog changed its function or expression level, it might have been advantageous to decouple its regulation by evolving a novel poison cassette exon in one paralog and releasing the selective pressure on its original poison cassette exon. The binding specificities of the three genes have also diverged during this time: SRSF4 has a GAAAA binding motif based on CLIP data (Änkö et al. 2012), whereas functional SELEX showed binding sites of TGCGTC for SRSF6 and TCACAGG for SRSF5 (Liu et al. 1998). Despite the minimal sequence required to create a poison cassette exon, the poison exon sequences in *SRSF4*, *SRSF5*, and *SRSF6* genes became remarkably constrained soon after they originated. The constraint could have arisen from binding sites for splicing regulators or features such as secondary structure; the sequence requirements for regulated splicing of these exons have not yet been dissected.

The mechanism and sequence constraint differ in fungi, whose *SRSF5* ortholog has an alternative retained intron at a conserved location but no poison cassette exon, while both forms are observed in metazoan *SRSF5*. It is possible that unproductive splicing of orthologs of *SRSF5*, in the same intron position, arose independently throughout the animal kingdom and also in fungi. However, given the extent of gene structure rearrangement between fungi and animals (Stajich et al. 2007; Csuros et al. 2011), the unusual conserved exon structure in this gene suggests selective pressure to maintain the unproductive splicing, although its regulation and function have not been explored in fungi. The short introns of fungal genes are generally spliced via intron definition, unlike long introns of metazoa (Romfo et al. 2000), and intron retention is prevalent in some fungi. Among *Aspergillus* species, the sequence of the retained intron is not notably conserved, suggesting a difference in regulation of unproductive splicing in fungi. Interestingly, the fungal intron that is retained is notably long, in the longest 1% of *Aspergillus* introns and the longest 3% of *Neurospora* introns, indicating that it has resisted the strong selective pressure toward shorter introns in fungi.

The evolution of unproductive splicing is a study in contrasting tempos: The ancient *SRSF5* unproductive splicing was conserved among all animals for hundreds of millions of years after the divergence of fungi, replaced rapidly in the *SRSF6* paralog in chordates, and then maintained in both forms—ancient and derived—for the full span of vertebrate evolution, under extreme sequence constraints. Unproductive splicing of *SRSF5* provides an exceptional instance of regulation by alternative splicing that is conserved in an entire kingdom and perhaps even conserved between orthologous introns in two kingdoms. Orthologous alternative splicing events at this distance have been considered improbable due to low conservation of alternative splicing within animals and due to the same process of turnover of splicing events that we report here (Lareau et al. 2004; Roy and Irimia 2009), although recently Awan et al. observed regulated alternative splicing shared between fungi and animals (Awan et al. 2013). Here, we show a detailed history, with numerous intermediate forms and modern descendents, of a conserved splicing event. That the ancient alternative splicing we demonstrate here is coupled to NMD raises questions about the ancestral role of alternative splicing. Across this evolutionary distance, the NMD mechanism itself has changed (Gatfield et al. 2003), yet its role in SR protein expression is conserved. This ancient yet evolutionarily dynamic system demonstrates flexible means to the same regulatory end.

## Materials and Methods

### Identification and Classification of Orthologs

Orthologs of *SRSF4*, *SRSF5*, and *SRSF6* were identified using existing annotations and sequence searches (supplementary tables S1 and S2, Supplementary Material online; Barbosa-Morais et al. 2006). In less-annotated species, genes with a high TBLASTN match to human SRSF4, SRSF5, or SRSF6 were aligned with all human SR genes, and phylogenetic trees were constructed to separate *SRSF4/5/6* orthologs from other SR proteins. The coordinates of some predicted genes were corrected based on EST alignments. To determine the relationship of all animal and fungus *SRSF4/5/6*-family genes, protein sequences were trimmed to the end of the second RNA recognition motif (RRM) domain and aligned along with an outgroup (*Arabidopsis* RSp31) using MUSCLE v3.7 (Edgar 2004). A maximum likelihood tree was built using proml and bootstrapped 100 times using seqboot from Phylip v3.68 (supplementary fig. S1*A*, Supplementary Material online; Felsenstein 1989). Low bootstrap values near the base of the tree led to uncertainties in the gene relationships. To further classify the genes, exon boundaries were mapped onto the protein sequence. Almost all genes had conserved exon boundaries clearly determining them to be *SRSF5*-like or *SRSF4/6*-like (supplementary fig. S1*B*, Supplementary Material online). Low bootstrap values prevented high-confidence classification of unspliced SR genes in sea squirt, lancelet, and anemone. ESTs showing alternative splicing or intron retention were found in UCSC and JGI genome browsers or, in fungi, by BLAST searches against ESTs in the NCBI nucleotide database.

### *Neurospora crassa* Splicing

Total RNA was extracted from *N. crassa* OR7A wildtype and *nam7*- strains (also known as NCU04242) using TRIzol reagent (Invitrogen). Five micrograms of *upf1-* RNA and 4.5 µg of wildtype RNA were reverse transcribed using SuperScript III (Invitrogen) with dT_15_ primers according to the manufacturer’s protocol. Two microliters of each 50-µl reverse transcription reaction was used in a PCR reaction with Phusion polymerase (New England Biolabs) following the manufacturer’s protocol, with a forward primer in exon 2 (CATCGAGTACAAGGATGCCA) and a reverse primer spanning the exon 4/5 junction (AACTCAACGAAGCCTTCACC). PCR products were run on a 2% agarose gel (supplementary fig. S1*C*, Supplementary Material online).

## Supplementary Material

Supplementary figures S1 and S2 and tables S1 and S2 are available at *Molecular Biology and Evolution* online (http://www.mbe.oxfordjournals.org/).

Supplementary Data
