# Supplementary material for: Regulation of Splicing Factors by Alternative Splicing and NMD Is Conserved between Kingdoms Yet Evolutionarily Flexible
Source: Mol Biol Evol. 2015 Mar 3;32(4):1072–9. doi: 10.1093/molbev/msv002 (PMC4379411; doi:10.1093/molbev/msv002)
Supplement: Supplementary Data [file supp_32_4_1072__index.html]

Regulation of Splicing Factors by Alternative Splicing and NMD Is Conserved between Kingdoms Yet Evolutionarily Flexible — Regulation of Splicing Factors by Alternative Splicing and NMD Is Conserved between Kingdoms Yet Evolutionarily Flexible — Supplementary Data 

# Regulation of Splicing Factors by Alternative Splicing and NMD Is Conserved between Kingdoms Yet Evolutionarily Flexible

## Supplementary Data

files

**Files in this Data Supplement:**

- Supplementary Data - pdf file
