# Supplementary material for: Regulation of Splicing Factors by Alternative Splicing and NMD Is Conserved between Kingdoms Yet Evolutionarily Flexible
Source: Mol Biol Evol. 2015 Mar 3;32(4):1072–9. doi: 10.1093/molbev/msv002 (PMC4379411; doi:10.1093/molbev/msv002)
Supplement: Supplementary Data [file supp_msv002_lareau_supporting.resub.pdf]

Supporting online material - Regulation of splicing factors by alternative splicing and NMD is conserved between kingdoms yet evolutionarily flexible

species	gene	genome source	locus	representative gene model or mRNA	alt splicing?	intron retention?	upstream junction ESTs	downstream junction ESTs	exon skipped ESTs	upstream junction intron retention	downstream junction intron retention	full intron retention	early polyA
H. sapiens	SRSF5	UCSC hg19	chr14:70233834-70238721	NM_006925	y	y	20	5	52	87	68	5	0
M. musculus	SRSF5	UCSC mm9	chr12:82046491-82051494	NM_009159	y	y	2	1	56	45	33	4	0
R. norgevicus	SRSF5	UCSC rn4	chr6:104728276-104732985	NM_019257	y	y	11	1	35	56	27	1	0
C. familiaris	SRSF5	UCSC canFam2	chr8:46549512-46554334	ENSCAFT00000026276		y	0	0	5	4	2	0	0
G. gallus	SRSF5	UCSC galGal3	chr5:30163796-30168433	NM_001031197	y	y	1	0	5	14	3	1	0
	SRSF5a	UCSC galGal3	chr5:30595571-30612641	NM_001006476	y	y	1	0	22	3	7	0	0
X. tropicalis	SRSF5	UCSC xenTro2	scaffold_246:1463821-1467713	NM_001004783	y	y	71	40	287	129	85	0	1
	krt5.1	UCSC xenTro2	scaffold_145:676974-689926	NM_001005638	y	y	1	0	30	1	2	0	0
F. rubripes	SRSF5b	UCSC fr2	chrUn:166973539-166976388	ENSTRUT00000045808			0	0	1	0	0	0	0
	SRSF5a	UCSC fr2	chrUn:270261895-270265478	ENSTRUT00000027205			0	0	0	0	0	0	0
D. rerio	SRSF5b	UCSC danRer5	chr20:38622161-38636988	NM_001002610	y	y	5	1	18	10	3	0	1
	SRSF5a	UCSC danRer5	chr13:38246638-38251475	NM_200867	y	y	11	4	176	43	9	1	0
B. floridae	SR A	UCSC braFlo1 / JGI v1.0	chrUn: 254615939-254624358	EST cluster 2601579:13	y		1	1?	76	1	0	0	0
	SR B	UCSC braFlo1 / JGI v1.0	chrUn:309654001-309657174	estExt_fgenesh2_pg.C_920027									
	SR C	UCSC braFlo1 / JGI v1.0	chrUn: 254826591-254835012	estExt_fgenesh2_pm.C_690014	y		1	1?	76	1	0	0	0
S. purpuratus	SR C	UCSC strPur2	Scaffold80892:724-9831	CX551350, CX557651	y		1	1	26	0	1	0	0
D. melanogaster	B52	UCSC dm3	chr3R:9487022-9492613	NM_176488	y	y	10	7	82	9	2	1	0
C. elegans	rsp-1	UCSC ce6	chrII:11453896-11455723	NM_064041	y	y	2	0	10	1	3	0	0
	rsp-2	UCSC ce6	chrII:11451360-11452871	NM_064040		y	0	0	15	1	1	1	0
N.vectensis	NV 1	JGI v1.0	scaffold_11:472975-477003	estExt_fgenesh1_pg.C_110055	y		3	4	18	0	0	0	0
	NV 2	JGI v1.0	scaffold_103:567405-570642	2667685_5	y		1	1	52	1	0	0	0
	NV 3	JGI v1.0	scaffold_318:144365-148353	estExt_fgenesh1_pg.C_3180016		y	0	0	11	0	1	0	0
	NV 4	JGI v1.0	scaffold_13:1331901-1334733	estExt_fgenesh1_pg.C_130169	y	y	3	1	0	0	2	0	0
	NV 5	JGI v1.0	scaffold_379:94033-94596	estExt_fgenesh1_pg.C_3790006									
	NV 6	JGI v1.0	scaffold_93:614755-619479	estExt_fgenesh1_pg.C_930062	y	y	3	2	20	0	1	0	0
N. crassa	NCU07069	Entrez Gene	NW_001849796.1:c267293-265451	XM_955241		y							
A. niger	An16g03890	Entrez Gene	NT_166531.1:c874159-871922	XM_001397663		y							
S. pombe	srp2	Entrez Gene	NC_003424.3:c4426965-4425250	NM_001019999									

Table S1 – SRSF5 orthologs included in analysis. Counts of ESTs and mRNAs showing alternative splicing are noted in the last columns (including partial evidence of intron retention, such as ESTs extending into the intron). We assigned temporary names to genes in C. intestinalis, B. floridae, S. purpuratus, and N. vectensis before phylogenetic classification

species	gene	genome source	locus	representative gene model or mRNA	alt splicing?	intron retention?	splicing in 2nd intron - upstream junction ESTs	splicing in 2nd intron - downstream junction ESTs	splicing in 2nd intron - exon skipped ESTs	splicing in 2nd intron - upstream junction intron retention	splicing in 2nd intron - downstream junction intron retention	splicing in 2nd intron - full intron retention	early polyA	splicing in 1st intron - upstream junction ESTs	splicing in 1st intron - downstream junction ESTs	splicing in 1st intron - exon skipped ESTs	splicing in 1st intron - upstream junction intron retention	splicing in 1st intron - downstream junction intron retention	splicing in 1st intron - full intron retention	early polyA
H. sapiens	SRSF4	UCSC hg19	chr1:29474251-29508637	NM_005626	y (i1, i2)	y (i2)	4	3	157	4	7	1	0	6	8	159	0	0	0	1
	SRSF6	UCSC hg19	chr20:42086504-42092242	NM_006275	y	y	58	6	132	10	9	0	0							
M. musculus	SRSF4	UCSC mm9	chr4:131429554-131457640	NM_020587	y (i1)	y (i1)	0	0	0	0	0	0	0	3	8	35	0	0	0	0
	SRSF6	UCSC mm9	chr2:162757244-162762854	NM_026499	y	y	2	19	94	3	19	1	0							
R. norgevicus	SRSF4	UCSC rn4	chr5:150731169-150758938	NM_001108685	y (i1)		0	0	0	0	0	0	0	1	1	3	0	0	0	0
	SRSF6	UCSC rn4	chr3:153795474-153800796	NM_001014185	y	y	1	3	23	0	14	0	0							
C. familiaris	SRSF4	UCSC canFam2	chr2:74298034-74323762	BU751463, DN425514 (gap in 1st intron)			0	0	0	0	0	0	0							
	SRSF6	UCSC canFam2	chr24:34189662-34192538	ENSCAFT00000014759, DN355700	y	y	1	1	2	0	1	0	0							
G. gallus	SRSF4	UCSC galGal3	chr23:3043852-3048526	BX936077, BX931065			0	0	0	0	0	0	0							
	SRSF6	UCSC galGal3	chr20:69285-74296	NM_001030843 (gap in intron)	y	y	0	3	13	0	3	0	0							
X. tropicalis	SRSF4	UCSC xenTro2	scaffold_492:434541-441727	CX803859	y (i2)	y (i2)	0	1	9	3	10	1	0							
	SRSF6	UCSC xenTro2	scaffold_38:805274-814512	NM_001001248	y	y	6	13	72	30	7	0	0							
F. rubripes	SRSF4	UCSC fr2	chrUn:139894565-139897692	ENSTRUT00000030178																
	SRSF6	UCSC fr2	chrUn:355227627-355231373	ENSTRUT00000007652	y		0	1	0	0	0	0	0							
D. rerio	SRSF4	UCSC danRer5	chr19:23988960-23998507	NM_199574		y (i2)	0	0	84	11	3	0	1							
	SRSF6a	UCSC danRer5	chr23:3858771-3870268	NM_213466			0	0	7	0	3	0	0							
	SRSF6b	UCSC danRer5	chr11:391396-400063	NM_001008732	y	y	1	2	11	4	3	0	0							
C. intestinalis	SR A	UCSC ci2	chr01q:6846290-6849708	AK116209	y*															
	SR B	UCSC ci2	chr01q:6888465-6893171	BW305692, BW324472	y*															
	SR C	UCSC ci2	chr02q:6965692-6967083	AK114189																
	SR D	UCSC ci2	chr05q:4307321-4308319	ESTs (unspliced in ORF)																
	SR E	UCSC ci2	chr05q:4319181-4321175	AK112385																

Table S2 – SRSF4 and SRSF6 orthologs included in analysis. As in table S1. “i1” indicates alternative splicing or intron retention in the first intron of SRSF4 orthologs, and “i2” indicates the second intron. All alternative splicing events in SRSF6 are in the second intron, and first-intron alternative splicing in SRSF4 was only observed in human, mouse, and rat, so many cells are not applicable and are left empty. Alternative splicing in C. intestinalis SR A and B was not homologous to alternative splicing in other species.

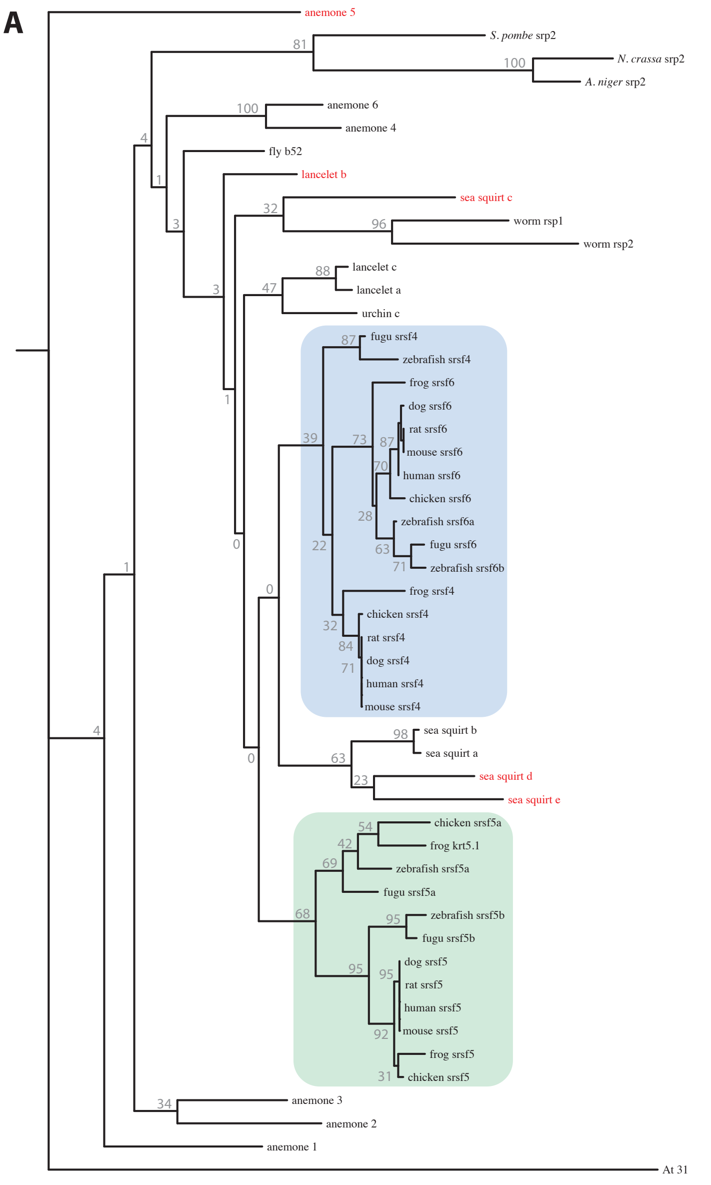

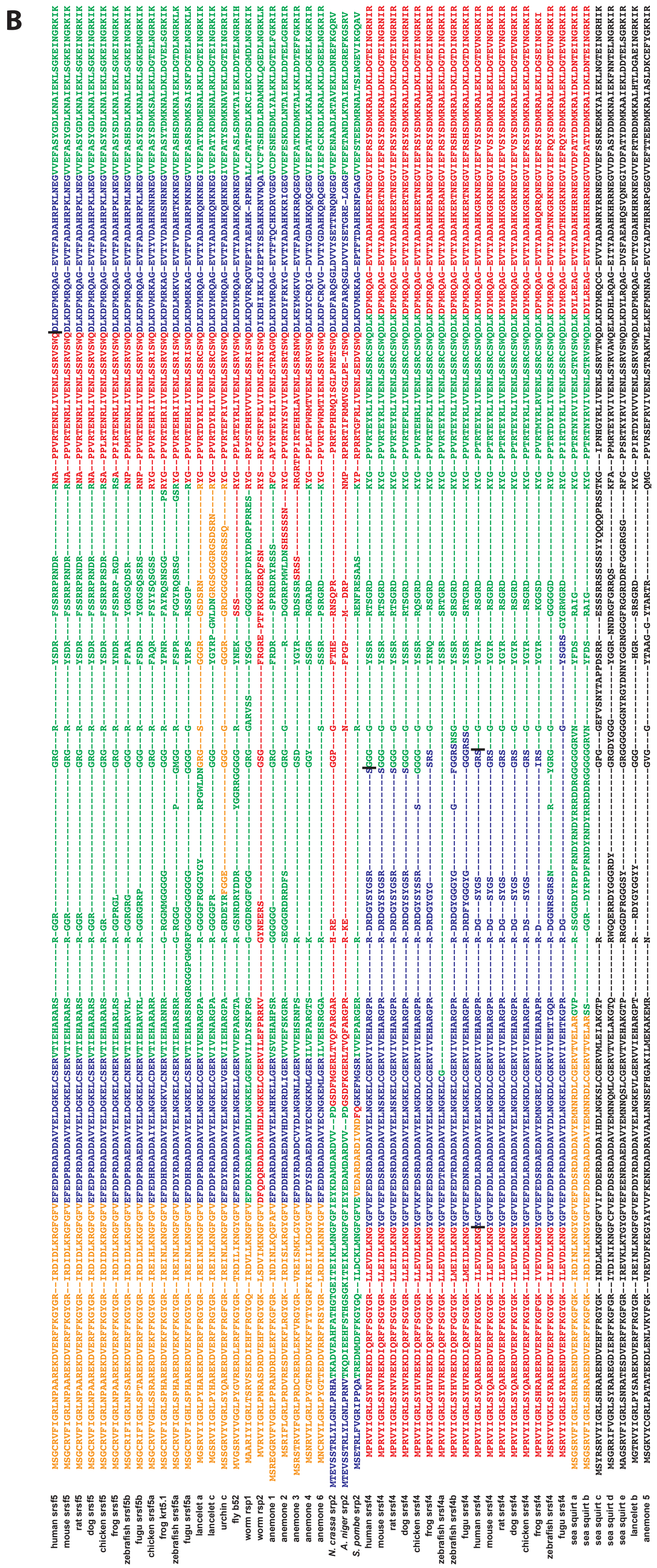

Figure S1 – A. Maximum likelihood tree of all animal and fungi SRSF4/5/6 genes, with bootstrap values. Vertebrate SRSF4/6 (blue) and SRSF5 (green) orthologs are grouped. Red indicates genes with no introns. B. Alignment of all SRSF4/5/6 protein sequences indicating exon positions. Colors denote exons. Sequences were trimmed after the second RRM domain before alignment. Black bars indicate the positions of alternative exons in human SRSF4, SRSF5, and SRSF6.

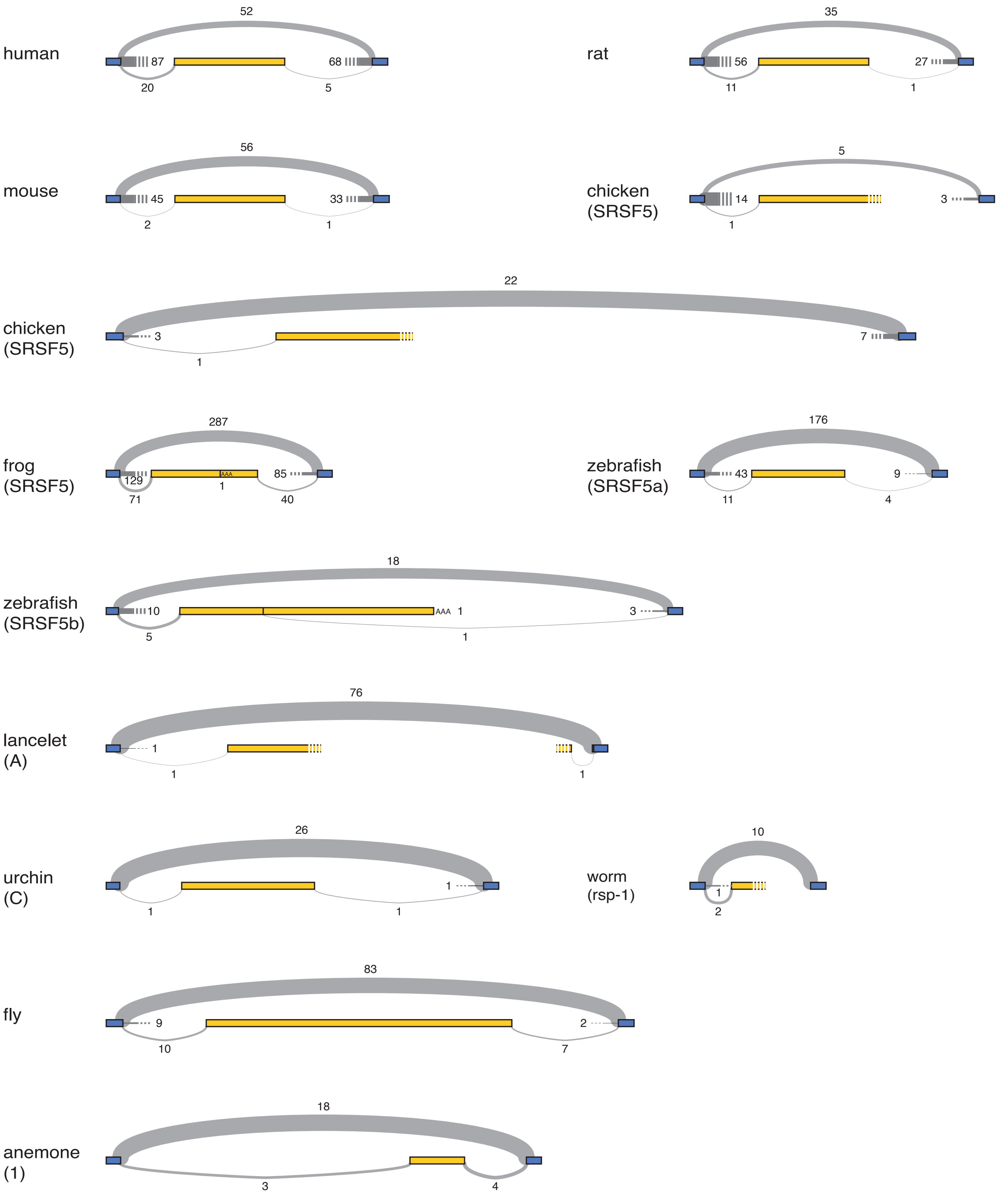

Figure S2 – Transcript evidence for alternative splicing of SRSF5. Alternative regions of each gene are shown with the upstream and downstream constitutive exons in blue and alternative exon in orange (to scale). Counts of ESTs and mRNAs showing alternative splicing are noted (including partial evidence of intron retention, such as ESTs extending into the intron) and splice junction lines are scaled to this count.
